# Redefining cerebral palsy: diagnostic boundaries based on the functional maturation of corticospinal motor control

**DOI:** 10.3389/fresc.2026.1936873

**Published:** 2026-09-28

**Authors:** Huaifeng Sun

**Affiliations:** Department of Child Rehabilitation, Huainan Maternal and Child Health Hospital, Huainan, Anhui, China

**Keywords:** accurate pincer grasp, cerebral palsy, corticospinal integration, developmental trajectory, final common pathway, stable independent walking

## Abstract

**Background:**

The 2006 international consensus defines cerebral palsy as a permanent disorder of movement and posture caused by non-progressive brain injury in the developing fetus or infant, yet it sets no upper age limit for the injury; in practice, countries adopt arbitrary chronological-age cut-offs, most commonly 24 months, without any neurobiological basis, and the 2025–2026 updated descriptions, although shifting toward a broader “description,” retain the same temporal framework and provide no operational diagnostic boundary.

**Core hypothesis:**

The diagnostic essence of cerebral palsy lies not in the chronological age at injury but in whether the injury occurs before the functional maturation threshold of corticospinal motor-control integration in the corresponding functional domain, producing a persistent deviation of that domain's motor developmental trajectory; the gross-motor domain is marked by stable independent walking and the fine-motor domain by accurate pincer grasp as an exploratory extension.

**Theoretical framework:**

Stable independent walking is the behavioural read-out of the first functional integration of spinal central pattern generators, brainstem postural control, vestibulo-cerebellar integration, and cortical descending modulation, with the corticospinal system as the final common pathway, so the framework applies to all motor subtypes; a four-criterion scheme justifies marker selection, and a complete operational definition is provided with velocity- and pattern-deviation domains, domain-specific criteria, and a stepwise diagnostic algorithm.

**Testable inferences:**

Stratification by integrative-maturation status is predicted to outperform chronological-age stratification in forecasting motor outcomes, with explicit falsification criteria and a multicentre prospective validation roadmap.

**Conclusions:**

Replacing the chronological-age criterion with the integrative-maturation status of each functional domain resolves the longstanding diagnostic ambiguity between cerebral palsy and other static motor impairments; all quantitative thresholds are theoretical working benchmarks pending calibration by multicentre prospective cohort studies.

## 1 Introduction

### 1.1 The core controversy: what exactly is a specific diagnostic criterion for cerebral palsy?

The 2006 international consensus statement and ICD-11 define cerebral palsy as a permanent disorder of movement and posture caused by non-progressive brain lesions in the fetus or infant (1–3). This definition has greatly advanced epidemiological surveillance and public-health policy-making, laying the foundation for globally unified standards in prevalence statistics, risk-factor screening, and allocation of medical resources (4, 5).

**Table 1:** The four operational criteria for a valid qualitative boundary marker of motor development.

Criterion	Operational definition
Non-linear shift in control strategy	Motor control shifts from one qualitative mode to another (e.g., spinal level → corticospinal synergy)
First multi-system integration	Multiple independently developing systems (CPG, cortex, vestibular, cerebellum) reach their functional threshold simultaneously
Developmentally irreversible	Once established, the pattern does not spontaneously regress to an earlier, lower-level form (distinguished from transient behaviours such as neonatal stepping); this does not imply immunity to later injury—injury can still damage the established pattern
Clinical identifiability	Can be reliably and objectively assessed in routine clinical settings

**Table 2:** Evaluation of the major motor milestones against the four-criterion framework for functional-boundary markers.

Milestone	Control-strategy shift	Multi-system integration	Irreversibility	Clinical identifiability	Qualifies
Head control (≈3 months)	No (mainly tone maturation)	No	Weak	Moderate	No
Independent sitting (≈6 months)	No (static posture)	No (no rhythm generation)	Moderate	High	No
Supported walking (≈10 months)	Partial (CPG activated, needs external support)	Partial	Weak	Moderate	No
Stable independent walking (≈12–15 months)	Yes (spinal → corticospinal synergy)	Yes (first full integration of CPG + cortex + vestibular + cerebellum)	Yes	Yes (including gait-pattern observation)	Yes (gross-motor domain)
Accurate pincer grasp (≈10–12 months)	Yes (proximal compensation → independent distal fine control)	Yes (first integration of tactile + visual + lateral CST)	Yes	Yes (including observation of compensatory movements)	Yes (fine-motor domain)*
Running (≈2 years)	No (a parametric variant of walking)	No	Weak	High	No
Jumping (≈2.5 years)	No (a derivative of the walking pattern)	No	Weak	High	No

* the “qualifies” judgement for the pincer-grasp row is based on indirect inference; its evidence strength is weaker than that of SIW. See Sections 2.1.2, 6.2.

**Table 3:** Pattern-deviation manifestations in the gross-motor and fine-motor domains.

Category	Gross-motor domain manifestation	Fine-motor domain manifestation
Abnormal tone	Spastic hypertonia of lower limbs/trunk	Hand flexor spasticity; distal hypotonia/dystonia
Fixed postural deformity	Equinus foot; persistent scissoring gait	Thumb-in-palm; wrist flexion contracture
Pathological reflexes	Persistent ankle clonus, positive Babinski sign	Persistent palmar grasp reflex; persistent ATNR
Abnormal motor patterns	Toe-walking compensation; scissoring; knee hyperextension; dystonic postural fluctuation	Three-finger-pinch compensation; shoulder/elbow elevation compensation; trunk leaning

**Table 4:** Combined diagnostic logic integrating velocity deviation and pattern deviation within each functional domain.

Velocity deviation	Pattern deviation	Clinical diagnostic conclusion
Present	Present	Cerebral palsy confirmed in that domain
Present	Absent	Isolated developmental delay
Absent	Present	Diagnose cerebral palsy only if injury occurred before integrative maturation and trajectory deviation persists
Absent	Absent	Cerebral palsy excluded in that domain

Note. The combination of velocity-deviation absent and pattern-deviation present (Row 3) applies primarily to two clinical scenarios: (i) pattern-deviation signs that do not directly invalidate the physiological-gait criterion for SIW attainment (e.g., persistent ankle clonus with well-compensated gait), and (ii) the fine-motor domain, where hand pattern-deviation (e.g., thumb-in-palm, wrist flexion contracture) coexists with independently attained gross-motor SIW. In the gross-motor domain, significant lower-limb pattern-deviation will typically cause failure of the physiological-gait criterion and thus present as velocity-deviation present rather than absent.

**Table 5:** Stepwise assessment grid for the integrative-maturation status and trajectory-deviation judgement of each functional domain.

Assessment step	Gross-motor domain	Fine-motor domain
Integrative-maturation status	Is SIW attained? (physiological gait)	Is pincer grasp attained? (de-compensated)
Result A	Yes → post-maturation; No → pre-maturation	Yes → post-maturation; No → pre-maturation
Trajectory-deviation judgement	Is velocity/pattern deviation present?	Is velocity/pattern deviation present?
Result B	Yes/No	Yes/No

However, this definition harbours a serious gap at the clinical level: it lacks explicit criteria for distinguishing cerebral palsy from other motor disorders that share the same non-progressive brain-injury aetiology yet present different clinical phenotypes. For example, a child who develops hemiplegia after traumatic brain injury at age 3 and a child with hemiplegia from perinatal stroke have entirely identical static brain lesions, yet their prognoses and rehabilitation programmes differ markedly—the current consensus definition cannot distinguish these two situations.

The notion of the “developing brain” in the consensus definition was supposed to carry this distinguishing function, restricting cerebral palsy to injuries that occur during the brain's period of active maturation; yet the term lacks any operational benchmark. Interpreted broadly, it would classify every static brain injury occurring before adulthood as cerebral palsy, stripping the diagnosis of its clinical meaning; interpreted narrowly, it relies entirely on subjective cut-offs—and this is precisely the root of the widespread clinical use of chronological-age thresholds. Because the consensus definition specifies no upper age limit, national surveillance systems and clinicians have arbitrarily set different thresholds, of which 24 months is the most commonly used. This age-based criterion exists purely for administrative convenience and lacks any neurobiological basis. The 2024 updated expert-panel interpretation still anchors the diagnosis to motor symptoms appearing before 24 months (6).

The proposed updated description of cerebral palsy published in 2025, although shifting the diagnostic wording from a “definition” to a “description” and incorporating new aetiological insights from genetics, inflammatory mechanisms, and neurophysiology, still retains the temporal framework of “early in development” and provides no operational boundary for distinguishing cerebral palsy from other static motor impairments (7); a commentary from the Surveillance of Cerebral Palsy in Europe likewise noted that this update remains ambiguous at the operational level (8). The updated description formally released in 2026 characterises cerebral palsy as “an early-onset, lifelong neurodevelopmental condition characterised by activity limitation,” further broadening its clinical-phenotype spectrum and whole-life-course perspective (9), but its temporal framework is unchanged—the phrase “occurring during early developmental stages” still cannot answer the central question of this paper: does a static brain injury that occurs in a child whose motor function has already largely matured belong to cerebral palsy? A Chinese expert consensus published the same year defines the time window of the causative brain injury as from the embryonic period to 3 years of age, adopts “no regression of previously acquired motor ability before age 5” as the clinical operational criterion for non-progression, and confirms genetic variants as important pathogenic factors (10). To our knowledge, this is the first consensus to pair an explicit upper age limit for the causative injury with a concrete, testable criterion for non-progression, and its recognition of genetic aetiology extends the pathogenic spectrum of cerebral palsy beyond classical perinatal brain injury. Two limitations, however, remain. First, the 3-year cut-off and the “before age 5” criterion are both expressed in chronological age, and the consensus therefore inherits the very age-centred boundary logic whose limitations are analysed in Section 3. Second, the non-regression criterion confirms only that the disorder is non-progressive; it does not discriminate between a persistent deviation of the developmental trajectory and an acquired loss of already-matured function, and it thus cannot answer whether a static brain injury sustained after motor maturation belongs to cerebral palsy—the central question of the present paper. Together, these developments show that the diagnostic “description” is becoming ever more precise, while the “boundary logic” that distinguishes cerebral palsy from other static motor impairments remains absent.

### 1.2 The core innovation of this hypothesis

This paper argues that the construct of the “developing brain” in the consensus definition harbours a critical conceptual ambiguity: it fails to distinguish whole-brain development from motor-function development—and it is the latter that is the core and defining domain of cerebral palsy. The fundamental reason the “developing brain” is unsuitable for diagnosing cerebral palsy is that the construct refers to whole-brain maturity, whereas the diagnostic core of cerebral palsy is the disruption of motor-function development caused by a specific brain injury; the two are essentially mismatched at the pathophysiological level.

Addressing this mismatch, this hypothesis proposes its central claim (full operational definition in Section 4.1): cerebral palsy is a group of syndromes caused by non-progressive brain injury and characterised by persistent deviation of the motor developmental trajectory; its diagnostic feature is that the injury occurs before the functional maturation threshold of corticospinal motor-control integration in the corresponding functional domain, thereby disrupting the construction of that domain's motor circuits. Each functional domain has its own behavioural marker of the critical threshold:

Gross-motor domain: marked by the attainment of stable independent walking (SIW), representing a state of functional maturation in which multi-system motor control is sufficient to execute the basic template of upright walking under a physiological gait pattern. “Physiological gait pattern” in this definition requires controllable ankle plantarflexion/dorsiflexion (no persistent toe-walking or foot drop), no excessive knee hyperextension or rigid locking during stance, and alternating hip flexion and extension (no persistent scissoring or abduction compensation).

Fine-motor domain: marked by the mastery of accurate tip-to-tip pincer grasp, representing a state of functional maturation in which hand motor control (particularly with participation of the lateral corticospinal system) is sufficient to perform the basic template of precise opposition without proximal joint compensation. “De-compensation” in this definition requires the wrist to remain in neutral or slight dorsiflexion (no flexion-contracture compensation), the forearm in neutral or slight supination (no excessive pronation compensation), and no compensatory elevation or abduction of the shoulder and elbow (no trunk-leaning compensation).

Two hierarchical levels must be clarified: the gross-motor domain is the core and the primarily testable part of this hypothesis, and the hypothesis can constitute a complete classification logic and be empirically tested on the gross-motor domain alone; the fine-motor domain is an exploratory extension—the status of accurate pincer grasp as a fine-motor developmental milestone has ample developmental-behavioural support, but its positioning as a “marker of the integrative-maturation critical threshold” currently rests on indirect inference, is weaker in evidence strength than SIW, and awaits direct testing by neurophysiological and multicentre studies (see Sections 2.1.2, 6.2).

The neurodevelopmental rationale for this hypothesis is that attainment of SIW marks the point at which the motor-control systems governing walking—the spinal central pattern generators, brainstem postural control, vestibulo-cerebellar integration, and cortical descending modulation—have first assembled into a stable, reproducible functional whole; attainment of accurate pincer grasp marks the point at which the control network governing distal fine hand actions has reached basic functional integration. It must be emphasised that the behavioural markers read the state of multi-system integration, not the “integrity” of the corticospinal tract as a single structure; the corticospinal system here plays the role of the final common pathway of voluntary movement (see Section 2.2). Before the corresponding marker is attained, these networks are under construction; once attained, their core structural architecture stabilises. Therefore: a brain injury occurring before the functional maturation threshold disrupts the network-building process of that domain, producing a permanent deviation of that domain's developmental trajectory; a brain injury occurring after the threshold damages an already matured functional network, causing loss of previously acquired motor ability but not altering the domain's original normal developmental-trajectory baseline.

The distinction between “persistent deviation of the developmental trajectory” and “loss of already acquired function” is highly consistent with clinical observation: a child with lifelong gait abnormality from perinatal stroke and a child who develops hemiplegia after traumatic brain injury following years of independent walking, although having identical static brain lesions, represent essentially different disease entities; a child with lifelong impairment of fine hand action from perinatal stroke and a child who loses fine hand function after brain trauma having previously mastered skilled manipulation likewise constitute essentially different clinical entities. The “physiological gait” and “de-compensated pincer grasp” criteria in this framework ensure diagnostic precision—the basis of diagnosis is not “whether a functional task can be completed” but “which neural circuit is used to complete it.” (Figure 1)

**Figure 1:**
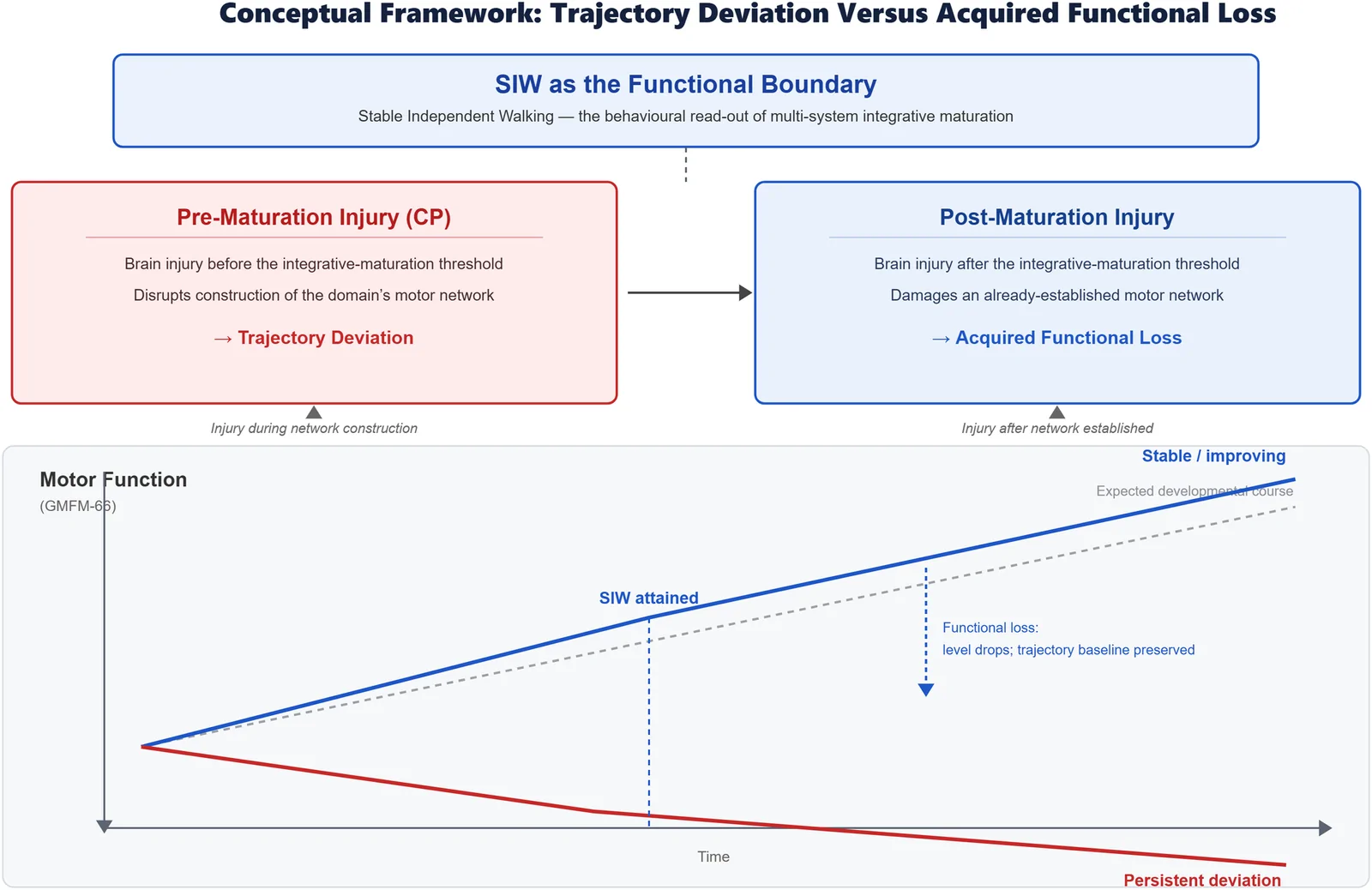
Conceptual framework of the integrative-maturation classification. Stable independent walking (SIW) is the behavioural read-out of the first functional integration of the spinal central pattern generators, brainstem postural control, vestibulo-cerebellar integration, and cortical descending modulation, with the corticospinal system as the final common pathway. A non-progressive brain injury occurring before the integrative-maturation threshold disrupts the construction of the domain's motor network, producing a persistent deviation of the motor developmental trajectory (cerebral palsy); an injury occurring after the threshold damages an already-established network, causing acquired functional loss without altering the original trajectory baseline.

### 1.3 The complementary relationship between this framework and existing classification systems

This hypothesis is complementary to, not a replacement for, existing classification tools:

GMFCS/MACS/CFCS: assess the functional severity of children already diagnosed with cerebral palsy, answering the question “how severe is the impairment” (11–13). This framework answers the antecedent diagnostic question “is it cerebral palsy.”

ICF: provides an overall model of disability assessment. This framework translates its abstract concepts into operational diagnostic practice (14, 15).

The 2006 consensus definition and the 2026 updated description: serve as the unified epidemiological and clinical descriptive baseline (1, 9). This framework merely substitutes the functional criterion of “functional maturation of corticospinal motor-control integration” for chronological age in individual clinical situations, so that the two criteria can be used in parallel; it can likewise run in parallel with the temporal boundary established by the Chinese expert consensus (10)—the former serves population surveillance and early-screening norms, while the latter serves individual diagnostic classification.

## 2 Neurodevelopmental theoretical foundation

### 2.1 The corticospinal system and the critical window of motor-circuit construction

#### 2.1.1 Gross-motor domain: the walking circuit

Upright walking depends on the coordinated maturation of multiple systems: corticospinal tract (CST) projections, spinal central pattern generators (CPG), brainstem postural control, and vestibulo-proprioceptive integration (16). After birth, lower-limb corticospinal projections undergo rapid developmental remodelling, whose active phase lies mainly within the first postnatal year and extends into the second, characterised by extensive synaptic pruning and strengthening of functional neural connections (17–21).

The emergence of SIW occurs within this remodelling window, marking that the foundational coordinative architecture on which walking depends—the synergy of spinal CPGs, brainstem postural control, vestibulo-cerebellar integration, and cortical descending modulation—has been assembled into a stable, reproducible motor pattern (22, 23). Thereafter, the walking circuit continues, within the same window, to fine-tune its parameters (such as step length and walking speed), rather than constructing the pattern *de novo*.

The positioning of SIW in this framework must be emphasised: SIW is the behavioural integrative read-out of the first functional integration of these multiple systems, not a marker of the maturation of the corticospinal tract as a single structure. The neural control of upright walking involves spinal rhythm generation, brainstem tonic postural maintenance, cerebellar timing and error correction, vestibular balance feedback, and cortical initiation, adaptation, and modulation of gait; these components are expressed, through the corticospinal system as the final common pathway of voluntary movement, as coordinated walking behaviour. This multi-system-integration positioning directly addresses the legitimate concern that “walking maturation is determined by factors beyond the corticospinal circuit”—this framework never reduces SIW to the integrity of a single pathway, but treats it as the terminal marker of the state of integration (Section 2.2 further argues its applicability to all motor subtypes).

#### 2.1.2 Fine-motor domain: the evidence base and hierarchical positioning of the pincer-grasp marker

Accurate tip-to-tip pincer grasp—the precise opposition of the thumb tip and index-finger tip—is a widely recognised milestone event in fine-motor development. Infant grasping follows a progressive sequence of “palmar grasp  →  radial grasp  →  finger-pad opposition (about 10 months)  →  tip-to-tip opposition (about 10–12 months).” This developmental fact is supported by extensive classical developmental-behavioural observation and standardised assessment tools (24–27), and is incorporated into the normative items of standardised scales such as the PDMS-2, the Bayley-III, and the Chinese Developmental Behavior Scale for Children Aged 0 to 6 Years (28–30).

On this basis, two qualitatively distinct claims must be strictly separated:

Claim A—“accurate pincer grasp is a recognised fine-motor developmental milestone”: this is a well-supported developmental fact in the literature, and this framework adopts it directly.
Claim B—“attainment of pincer grasp marks the functional maturation threshold of fine-motor corticospinal integration”: this is a new theoretical construct of the present hypothesis. In currently published literature, direct experimental evidence for this specific proposition is lacking. The series of studies by Forssberg and colleagues shows that anticipatory force-control strategies for precision grasping continue to mature toward the adult form throughout childhood and even adolescence (26, 27); transcranial magnetic stimulation studies by Fietzek and colleagues likewise confirm that central conduction maturation of the corticospinal system spans the whole of childhood (31). However, these studies focus on “how finely grasping is regulated” (continued fine-tuning of anticipatory control strategies), not on “whether the opposition movement can be independently initiated” (the functional establishment of the basic motor template); the two differ essentially in conceptual level, and the former cannot be used to refute the latter.

Therefore, the justification for Claim B in this hypothesis rests on the following indirect inferences: (1) at the first appearance of pincer grasp, the excitatory conduction pathway from the corticospinal tract to the cervical motoneuron pools must already possess basic anatomical connectivity (a necessary condition); (2) the stable execution of this movement requires completion without proximal compensation, ruling out reliance on spinal reflexes or the propriospinal brainstem-spinal pathways alone, and thereby implying that cortical descending modulation already participates; (3) once stably established, this movement does not spontaneously regress to the earlier radial-grasp pattern, suggesting that the core circuit architecture has stabilised.

Accordingly, this framework adopts the following hierarchical positioning for the fine-motor domain: the gross-motor domain (SIW) constitutes the core hypothesis, and this hypothesis can be fully established and tested on the gross-motor domain alone; the fine-motor domain is an exploratory extension. The positioning of pincer grasp as the boundary marker is a theoretical hypothesis with a reasonable behavioural-developmental basis but still awaiting neurophysiological verification; its evidence strength is explicitly weaker than that of SIW, and it is listed as the primary limitation of this framework (see Section 6.2). The distinction between Claim A and Claim B ensures that even if future research refutes Claim B, the core classification logic of the gross-motor domain remains entirely unaffected.

#### 2.1.3 A four-criterion framework for functional-boundary markers

To rigorously justify choosing SIW and pincer grasp as the diagnostic boundary markers of their respective functional domains, this hypothesis proposes that a valid “qualitative boundary” of motor development must simultaneously satisfy four operational criteria (32, 33):

Applying this four-criterion framework to the major motor milestones:

In summary, among the key motor milestones, SIW and accurate pincer grasp simultaneously satisfy all four criteria, serving respectively as the functional-maturation markers of the gross- and fine-motor domains; the evidence base for SIW is robust, whereas that for pincer grasp awaits verification.

### 2.2 Applicability to all motor subtypes: the final-common-pathway argument

One question must be addressed head-on: this framework takes “corticospinal integration” as its theoretical core, so are the cerebral-palsy subtypes whose primary lesion is not the corticospinal tract—the dyskinetic type (mainly basal-ganglia injury), the ataxic type (mainly cerebellar injury), and the hypotonic type—also within its scope of applicability? This section argues that the answer is yes, and that no modification of the framework is required.

The argument proceeds in three steps. First, the corticospinal system is the final common pathway of all voluntary movement: the basal ganglia and the cerebellum do not bypass this pathway in their output; their motor-modulatory functions converge, respectively, via thalamo-cortical projections (the basal-ganglia loop) and dentato-thalamo-cortical projections (the cerebellar loop), onto the motor cortex, and are then executed through the descending corticospinal tract. Therefore, injury to the basal ganglia or the cerebellum in fact manifests as abnormal motor control on the final common pathway of corticospinal integration, by interfering with the generation and shaping of cortical motor commands. Second, SIW and pincer grasp, as behavioural markers, read the state of integration of the entire motor network (Section 2.1.1), not the integrity of the corticospinal tract as a single structure—so long as the construction of network integration is disrupted, whether the site of disruption lies in the cortex, the basal ganglia, or the cerebellum, the behavioural read-out will show deviation. Third, the diagnostic basis of this framework is “the presence of trajectory deviation,” not “the inferred site of the lesion”; therefore, subtype-specific differences in pathological mechanism do not affect the decision logic.

#### By subtype

##### Dyskinetic type

Dystonia and involuntary movements are explicitly included in the pattern-deviation criteria (Section 4.2). Even if such children eventually attain the ability to walk independently, their dystonic gait (fluctuating tone, unstable postural control) necessarily fails to meet the “physiological gait pattern” criterion for SIW, and they are judged as having incomplete integrative maturation of the gross-motor domain.

##### Ataxic type

Cerebellar injury causes markedly delayed independent walking (often beyond 24 months), constituting velocity deviation; a wide-based gait, dysmetria, and intention tremor constitute pattern deviation. Notably, the cerebellum is itself a component of the “first multi-system integration” of SIW in the four-criterion framework (Section 2.1.3), so the disruption of the integrative process by cerebellar injury has a direct mechanistic correspondence within this framework.

##### Hypotonic type

This is the diagnostically most challenging subtype, because infantile hypotonia is highly confounded with primary neuromuscular disease, genetic metabolic disease, and benign congenital hypotonia. The handling by this framework is in fact clearer than the current consensus: the boundary clause in Section 7 already excludes primary musculoskeletal and peripheral neuromuscular diseases; on that premise, the retained primitive reflexes, abnormal postural reactions, and delayed motor milestones that accompany central hypotonia can still be detected through both pattern deviation and velocity deviation. Hypotonic cerebral palsy is essentially an exclusion diagnosis, and the exclusion clause of this framework happens to provide a clear boundary for that exclusion.

##### A candid caveat is in order

The trajectory-deviation patterns of different lesion-site subgroups may differ—for example, the ataxic type may manifest mainly as velocity deviation, whereas the dyskinetic type manifests mainly as pattern deviation. The deviation characteristics of each subgroup and their effect on diagnostic sensitivity should be analysed as independent subgroups in validation studies (Section 8).

Building on the above evidence, Section 4 formalises this theoretical foundation into an operational diagnostic definition centred on integrative maturation; the functional advantages and clinical implications of this definition are detailed in Section 6.1.

## 3 Chronological age cannot adequately substitute for motor maturity

### 3.1 The value and limitations of the 24-month cut-off

This paper explicitly opposes abolishing the 24-month chronological-age criterion. It is indispensable in population surveillance, longitudinal public-health tracking, and cross-national comparison of prevalence data (4), and the Chinese expert consensus likewise incorporates it into the temporal framework of early-diagnosis norms (10). However, population statistical benchmarks and individual diagnostic criteria must be strictly separated: retain the 24-month criterion for population surveillance and early screening, while adopting the functional criterion of corticospinal integrative maturation only in individual clinical rehabilitation care.

### 3.2 Representative clinical scenarios

Using chronological age as the diagnostic boundary marker produces two types of diagnostic error:

Scenario A (false positive): a child attained SIW (with a physiological gait) at 15 months, and then developed hemiplegia after traumatic brain injury at 23 months. By the 24-month criterion the child is diagnosed with cerebral palsy. Yet functionally, the walking template had already fully matured, and the injury merely caused an acquired loss of an already established function. The chronological-age criterion produces a false-positive diagnosis.
Scenario B (false negative): a child never attained SIW before 24 months, and then sustained a static brain injury causing hemiplegia at 25 months. By the 24-month criterion, the injury occurred outside the “window,” and cerebral palsy is excluded. In fact, however, the walking template was never established, and the injury directly disrupted an actively developing motor network, fully meeting the functional definition of cerebral palsy. The chronological-age criterion produces a false-negative diagnosis.

### 3.3 The urgency of establishing a functional boundary criterion

The root of these clinical challenges lies in mistakenly equating chronological age with neuromotor maturity. The solution is to adopt validated functional biomarkers—the functional maturation of corticospinal motor-control integration in each domain—with SIW under a physiological gait pattern and de-compensated accurate pincer grasp as the operational markers, embedded within standardised motor-assessment frameworks (GMFM, PDMS-2) (28, 34).

## 4 A theoretical framework based on the functional maturation of corticospinal motor-control integration

### 4.1 Operational definition of cerebral palsy (core hypothesis)

Cerebral palsy is a group of syndromes caused by non-progressive brain injury and characterised by persistent deviation of the motor developmental trajectory; the injury occurs before the functional maturation threshold of corticospinal motor-control integration in the corresponding functional domain, disrupting the construction of that domain's motor circuits. Each functional domain has its own behavioural marker of the threshold: the gross-motor domain is marked by the attainment of stable independent walking (SIW) (the core adjudicating domain); the fine-motor domain is marked by the mastery of accurate pincer grasp (an exploratory extension). Trajectory deviation in any functional domain, combined with an injury occurring before the functional maturation threshold of that domain, suffices for a diagnosis of cerebral palsy.

This definition contains four requisite elements:

Non-progressive brain injury—the pathological basis;
Injury occurring before the integrative-maturation threshold of the corresponding functional domain—a temporal criterion, judged domain by domain;
Persistent deviation of the motor developmental trajectory of the corresponding functional domain—a functional outcome, judged domain by domain;
The judgements of the gross- and fine-motor domains are mutually independent—meeting the criterion in any single domain suffices for diagnosis.

It should be noted that “corticospinal integration” in this definition is understood in its multi-system-integration sense (Section 2.2): the corticospinal system, as the final common pathway of voluntary movement, carries and expresses the integrative result of a network jointly involving the cortex, basal ganglia, cerebellum, brainstem, and spinal cord; therefore the definition covers all motor subtypes of cerebral palsy.

### 4.2 Two operational quantitative domains for identifying persistent developmental-trajectory deviation

Domain 1: velocity deviation (quantitative criterion) (35).

Gross-motor domain: failure to attain SIW under a physiological gait pattern by a corrected age of 24 months.

Fine-motor domain: inability, at a corrected age of 18 months, to stably complete accurate pincer grasp without proximal compensation.

Domain 2: pattern deviation (qualitative criterion).

Manifesting as abnormal tone, fixed postural deformity, pathological reflexes, and abnormal motor patterns, which must persist across two assessments separated by 1 to 2 months. The judgement of abnormal muscle tone adopts clinical qualitative description (spastic hypertonia, hypotonia, dystonia) and does not preset any scale cut-off value—any quantitative cut-off is an uncalibrated theoretical setting, and its calibration is listed as the first task of validation research (Section 8). It is particularly worth noting that the dynamic component of mild spasticity (grade 1 or 1+ on the Modified Ashworth Scale) can already cause gait deviation, and that such deviation may be masked by compensatory mechanisms (36, 37); therefore this framework does not rely on resting-state muscle-tone grading, but instead captures it through the “physiological gait pattern” criterion of Section 4.3.1 (including observation of persistent toe-walking, knee hyperextension, and scissoring) combined with systematic observational gait analysis (OGA)—an individual with such a dynamically abnormal gait will be judged as failing SIW criterion 3, i.e., integrative maturation is incomplete.

Combined diagnostic logic (applied independently to each functional domain):

Overall diagnostic conclusion: if any functional domain satisfies “velocity deviation + pattern deviation present,” and the injury occurred before the integrative-maturation threshold of that domain, cerebral palsy can be diagnosed.

Special determination for severe impairment: children with severe impairment (GMFCS level IV/V) who never attained SIW, and children with severe hand spasticity who never attained accurate pincer grasp, although lacking the corresponding overt behavioural marker, clearly have impaired corticospinal integrative maturation; this can be judged through surrogate indicators such as persistent primitive reflexes, abnormal tone, and abnormal posture, and still falls within the diagnostic scope of the corresponding functional domain (11, 12).

### 4.3 Operational diagnostic criteria for the markers of each functional domain

#### 4.3.1 Gross-motor domain: operational diagnostic criteria for SIW

Confirmed by two independent assessments at least one week apart, and simultaneously meeting the following:

Functional autonomy: independently initiating, continuing to walk at least 5 metres without assistance, and stopping autonomously;
Dynamic balance: completing a 90-degree turn during walking without external support and without falling;
Physiological motor pattern: controllable ankle plantarflexion/dorsiflexion (no persistent toe-walking or foot drop), no excessive knee hyperextension or rigid locking, and alternating hip flexion and extension (no persistent scissoring).

SIW assessment should be embedded within standardised gross-motor assessment frameworks (GMFM, PDMS-2) rather than used as an isolated observational item (28, 34). To avoid misunderstanding, we clarify that the three-component operational criterion above (5-metre walk, 90° turn, qualitative gait-pattern judgement) is an author-defined combination of individual items drawn from these standardised instruments—for example, the standing and walking items of the GMFM and the locomotion and balance subscales of the PDMS-2—together with a qualitative judgement of gait pattern; none of the cited scales provides this composite SIW criterion as a built-in standard. The role of the standardised tools is to supply the item pool, the age-referenced norms, and the structured assessment context within which this criterion is applied. Judgement of gait pattern is recommended to be combined with systematic observational gait analysis (OGA) so as to identify dynamic abnormalities masked by compensatory mechanisms (36, 37).

#### 4.3.2 Fine-motor domain: operational diagnostic criteria for accurate pincer grasp

Confirmed by two independent assessments at least one week apart, and simultaneously meeting the following:

Independent tactile-motor initiation: precise opposition of the thumb tip and index-finger tip to retrieve a 5 mm pellet;
Repetitive stability: completing at least three consecutive retrievals in one sitting test;
De-compensated motor pattern: wrist in neutral or slight dorsiflexion, forearm in neutral or slight supination, no compensatory shoulder/elbow elevation or abduction, and no trunk leaning.
Decision note: if the pellet can only be lifted with a three-finger pinch or radial grasp, or if it is accompanied by shoulder elevation or trunk leaning, the integrative maturation of the fine-motor domain is judged to be incomplete. Both sides should be assessed; if one side is severely affected, stable completion on the unaffected side is taken as the behavioural marker, while the affected side is evaluated for its degree of deviation.

Note on quantitative thresholds: the above parameters are theoretical working benchmarks based on existing standardised assessment tools (28–30, 34); the optimal diagnostic thresholds await calibration by multicentre prospective studies through ROC curves.

### 4.4 A complete stepwise clinical diagnostic algorithm

Stage 1: confirmation of the shared premise—is a non-progressive brain injury confirmed?

Stage 2: independent parallel assessment of each functional domain

For children assessed a considerable time after the injury, the integrative-maturation status at the time of injury is determined retrospectively on the basis of medical history, neuroimaging, and prior assessment records.

Stage 3: integrated diagnostic classification

Cerebral palsy confirmed: any functional domain satisfies “pre-maturation + trajectory deviation present”;

Cerebral palsy excluded: neither functional domain satisfies the above condition;

Mixed classification: where the two domains are classified differently, each is stated separately;

Under observation: where the status of any domain is unclear, a 3–6 month observation window is initiated. (Figure 2)

**Figure 2:**
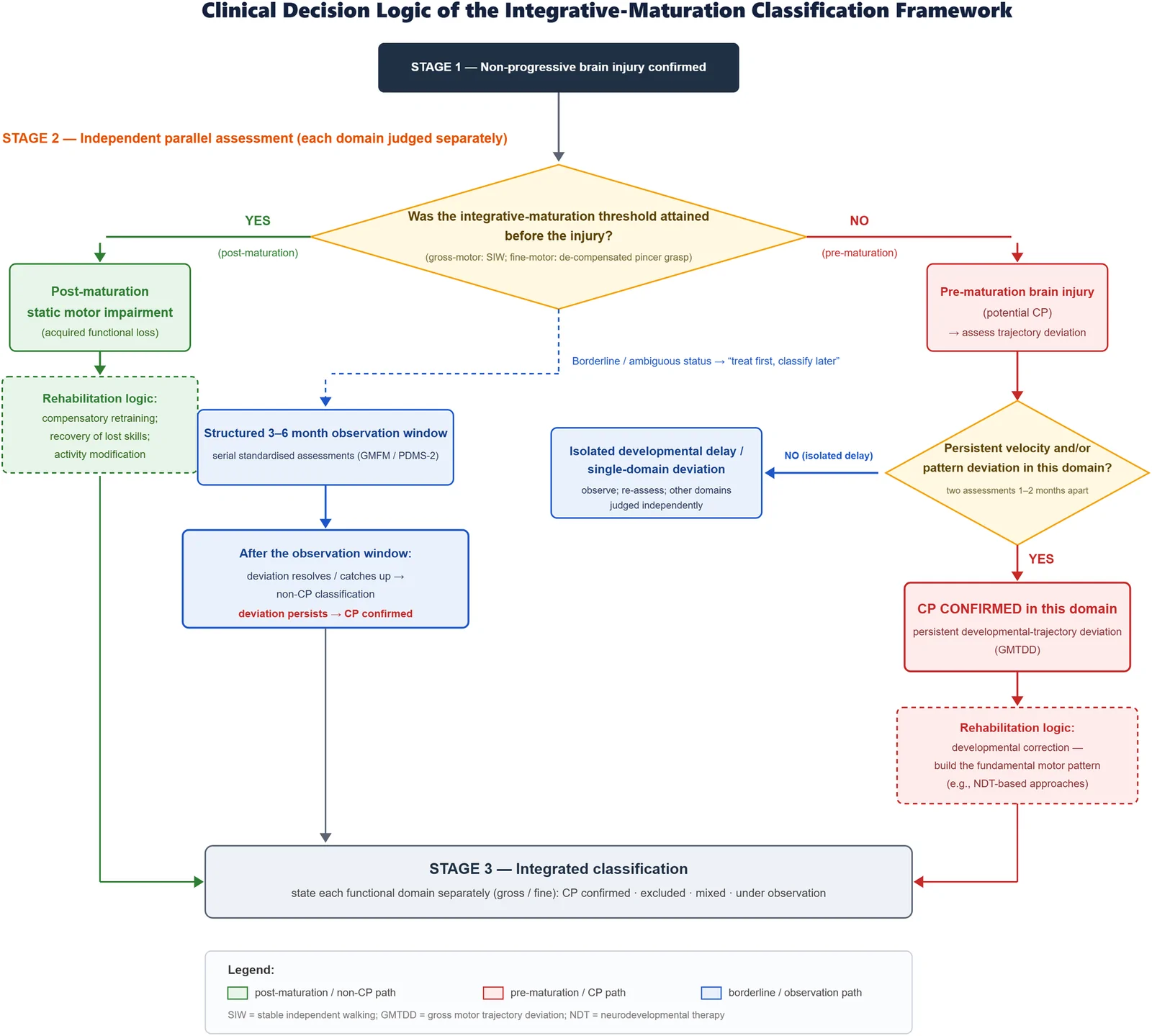
Clinical decision logic of the integrative-maturation classification framework. Each functional domain is judged independently. The framework first confirms a non-progressive brain injury (Stage 1), then assesses, for each domain, the integrative-maturation status at the time of injury and the presence of persistent velocity and/or pattern deviation (Stage 2), and finally integrates the domain-specific results into an overall classification (Stage 3: confirmed, excluded, mixed, or under observation). Borderline or ambiguous cases follow a structured 3–6 month observation window with serial standardised assessments. SIW, stable independent walking; GMTDD, gross motor trajectory deviation; NDT, neurodevelopmental therapy.

## 5 The core hypothesis and empirically testable inferences

### 5.1 Four quantifiable empirical inferences

Inference 1: discriminant validity. Three-year longitudinal follow-up will show that persistent motor-developmental-trajectory deviation exists only in children with cerebral palsy whose brain injury occurred before integrative maturation; children whose brain injury occurred after integrative maturation (such as childhood arterial ischaemic stroke or traumatic brain injury) will, after the acute-phase functional loss, show a stable or improving motor trajectory (38, 39), rather than persistent trajectory deviation. Existing studies of motor-developmental curves in cerebral palsy (40, 41) provide direct evidence for the trajectory characteristics on the cerebral-palsy-cohort side; on the “post-maturation-injury trajectory tends to stabilise or improve” side, currently only recovery-trajectory studies of childhood stroke cohorts provide preliminary indirect support (38, 39), which is precisely the core content to be tested by this inference. Falsification criterion: no statistically significant difference in trajectory pattern between the two cohorts.

Inference 2: predictive validity. Stratification by the integrative-maturation status of each functional domain predicts 3-year functional-assessment scores better than chronological-age grouping. Falsification criterion: ROC analysis shows that chronological-age stratification predicts equally well or better than integrative-maturation stratification.

Inference 3: rehabilitation-programme matching efficacy. Children who receive a rehabilitation programme matched to the integrative status at the time of injury show significantly greater functional gains than children who receive a mismatched intervention (42–45). Falsification criterion: no significant interaction between programme matching and functional outcome.

Inference 4: medical-resource optimisation benefit. Stratification based on integrative-maturation status reduces unnecessary specialist referrals and shortens ineffective rehabilitation cycles. Falsification criterion: integrative-maturation stratification neither reduces cost nor improves diagnostic precision.

## 6 Discussion

### 6.1 The advantages of this framework

A solid neurodevelopmental scientific foundation, supported by multi-system-integration and final-common-pathway theory, and consistent with the developmental timelines of the respective neural pathways;

Applicability to all motor subtypes of cerebral palsy: regardless of whether the lesion lies in the corticospinal tract, the basal ganglia, or the cerebellum, it is detected through the behavioural read-out of the final common pathway (Section 2.2);

Each functional domain is judged independently, with “physiological pattern/de-compensation” as the precondition for each, and deviation in any single domain suffices for diagnosis;

The assessment criteria can be completed in primary-care settings by clinical observation, without advanced equipment;

It accommodates the inter-individual variability of motor development;

It directly guides the design of domain-targeted rehabilitation programmes.

### 6.2 Limitations

A purely theoretical construct, lacking original prospective clinical data;

The quantitative diagnostic thresholds are theoretical assumptions, not calibrated by prospective clinical trials;

The fine-motor domain is an exploratory extension: the status of accurate pincer grasp as a fine-motor developmental milestone has ample developmental-behavioural support, but its positioning as a “marker of the integrative-maturation critical threshold” currently rests on indirect inference and lacks WHO-level standardised norms for the attainment window (46) (evidence strength weaker than SIW); this is the greatest shortcoming of this paper, and the neurobiological validity of this marker awaits direct testing by subsequent multicentre prospective studies; the distinction between Claim A (milestone status) and Claim B (threshold-marker status) ensures that the core hypothesis is unaffected by this shortcoming;

For children at GMFCS level IV/V and those with severe hand spasticity, the judgement relies on surrogate indicators;

External validity across different demographic and ethnic cohorts has not been validated; the differences in trajectory-deviation patterns among the motor subtypes still require subgroup data to elucidate;

Serial functional assessments may increase the clinical workload;

The “functional-domain threshold paradigm” of this framework applies only to the gross- and fine-motor circuits of the limbs. Oropharyngeal movement is mediated by cortico-bulbar-brainstem circuits and lacks a behavioural critical marker that satisfies the four-criterion framework of this study; therefore oropharyngeal function cannot serve as a diagnostic adjudicating domain of cerebral palsy (47, 48).

### 6.3 Implications for clinical-trial design

Future clinical trials of cerebral-palsy rehabilitation should use stratified randomisation by the affected functional domain, taking the integrative-maturation status of each domain at the time of injury as the stratification variable (42–44).

### 6.4 Benefits to each stakeholder

Clinicians: unified and explicit diagnostic criteria, directly guiding individualised rehabilitation planning;

Caregivers: domain-specific, objective prognostic counselling;

Healthcare administrators: rational allocation of limited rehabilitation resources;

Policy-makers: a parallel dual-criterion system—the chronological-age criterion serving population surveillance, and the functional criterion serving individual diagnosis.

## 7 Boundary conditions and comorbidities

This framework is designed specifically for classifying motor disorders caused by non-progressive brain injury, and does not apply to primary musculoskeletal disease or peripheral neuromuscular disease. The diagnoses of the gross- and fine-motor functional domains are mutually independent; clinical reports should state the affected functional domain(s) and the integrative-maturation status at the time of injury. As noted earlier (Section 2.2), this boundary clause simultaneously provides a clear operational basis for the exclusion diagnosis of hypotonic cerebral palsy: once hypotonia caused by primary neuromuscular disease is excluded, central hypotonia can still be detected through both pattern deviation and velocity deviation.

Oropharyngeal motor abnormalities (swallowing, respiratory-vocal coordination) are not used to trigger a cerebral-palsy diagnosis in this framework. However, for individuals already diagnosed with cerebral palsy, oropharyngeal incoordination is an important comorbidity, requiring separate specialist assessment of swallowing and speech and inclusion in a comprehensive rehabilitation plan (47, 48). Diseases whose core manifestation is oropharyngeal impairment (such as congenital bulbar palsy) follow specialist diagnostic systems and fall outside the scope of this framework.

## 8 Roadmap for empirical validation research

### 8.1 Primary core objective

A multicentre prospective controlled cohort study to validate the predictive validity of SIW classification (gross-motor domain, core hypothesis) in head-to-head comparison with traditional chronological-age stratification; pincer-grasp classification (fine-motor domain, exploratory extension) is included in the same cohort as a secondary validation objective.

### 8.2 Supporting objective 1a: clinical calibration of quantitative diagnostic thresholds

Calibrate the assessment criteria for SIW and pincer grasp, and determine the optimal diagnostic cut-off for each domain through ROC curve analysis. Among these, the velocity-deviation threshold at 18 months for the fine-motor domain has the weakest evidence base and the highest calibration priority.

### 8.3 Supporting objective 1b: age-specific diagnostic thresholds and compensation-severity grading

Establish age-stratified norms of SIW diagnostic parameters, and develop a Compensation Severity Scale (CSS). At the same time, conduct independent subgroup analyses by motor subtype (spastic, dyskinetic, ataxic, hypotonic) to elucidate the differences in trajectory-deviation patterns among subtypes and their effect on diagnostic sensitivity.

### 8.4 Supporting objective 2: development of standardised assessment tools

Develop an operational clinical-practice manual, a screening scale for primary-care settings, and a telemedicine assessment protocol.

### 8.5 Supporting objective 3: implementation science and health-economic evaluation

Compare the cost-effectiveness of early integrative-maturation stratification vs. traditional delayed age-based classification.

## 9 Conclusion

Contemporary diagnostic definitions of cerebral palsy rely on the vague construct of the “developing brain,” which in routine clinical practice is reduced to rigid chronological-age cut-offs, resulting in widespread diagnostic ambiguity. This paper proposes an empirically testable solution:

The diagnostic boundary of cerebral palsy lies not in the chronological age at injury, but in the relationship between injury and functional maturation—an injury before integrative maturation disrupts the construction of the motor pattern in the corresponding functional domain, producing a persistent deviation of the developmental trajectory; an injury after integrative maturation merely causes an acquired loss of the established pattern without altering the original trajectory baseline. This distinction establishes a clear diagnostic boundary between cerebral palsy and other static motor impairments caused by non-progressive brain injury.

The core content of this framework is summarised in the following key points:

Operationalisation of the diagnostic boundary: the gross-motor domain is marked by stable independent walking under a physiological gait pattern, and the fine-motor domain by de-compensated accurate pincer grasp; each domain is judged independently, and meeting the criterion in any single domain suffices for diagnosis;

The chronological-age criterion produces two identifiable diagnostic errors: false positives for injuries at the tail end of the pre-maturation window (Scenario A) and false negatives for immature children outside the window (Scenario B);

The framework covers all motor subtypes of cerebral palsy through the final-common-pathway argument;

All quantitative thresholds are theoretical working benchmarks pending calibration.

We clearly anticipate that future large-sample calibration studies will advance: (1) the establishment of age- and height-specific percentile curves for physiological gait parameters; (2) the establishment of a compensation-severity gradient assessment system; and (3) the determination of the optimal values of each quantitative cut-off through ROC analysis. This open design of continuous optimisation based on empirical feedback is precisely the essential feature that distinguishes this framework from existing subjective-cut-off systems—it is a falsifiable, revisable, and calibratable classification system. Its ultimate value awaits the testing and refinement of future clinical research and practice.

## Data Availability

The original contributions presented in the study are included in the article/Supplementary Material, further inquiries can be directed to the corresponding author/s.
